# Preparation and Tumor Inhibitory Activity of Tricin from Carex Meyeriana Kunth

**DOI:** 10.3390/molecules29194530

**Published:** 2024-09-24

**Authors:** Baiji Cui, Jie Sun, Sheng Chang, Hongmei Zhang, Yawei Li, Xianmin Feng, Zengjun Guo

**Affiliations:** 1 School of Pharmacy, Health Science Center, Xi’an Jiaotong University, Xi’an 710061, China; baijicui_jlmu@163.com; 2 School of Pharmacy, Jilin Medical University, Jilin 132013, China; changsheng-pharm@hotmail.com (S.C.); hongmeizhang_1981@163.com (H.Z.); lyw135@163.com (Y.L.); 3 School of Basic Medicine Sciences, Jilin Medical University, Jilin 132013, China; sunjie1014@163.com

**Keywords:** Carex Meyeriana Kunth, HPLC, macroporous adsorption resins, tricin, tumor

## Abstract

This study describes the purification and preparation of tricin (5, 7, 4-trihydroxy-3, 5-dimethoxyflavone) from Carex Meyeriana Kunth via adsorption and desorption using macroporous resins and high-performance liquid chromatography. Six resins were tested to evaluate the static adsorption and desorption capacities. The HPD-300 resin was selected as the adsorption material to enrich tricin because of its suitable adsorption and desorption capacities. Adsorption thermodynamics and kinetics were studied on HPD-300 resin, and the results agreed with the Langmuir model and quasi-second-order kinetics model, respectively. The parameters of the dynamic adsorption and desorption tests were then optimized. The purity of tricin increased from 2.6 mg/g to 45.1 mg/g with a recovery yield of 76.4% after purification using HPD-300 resin. Then, Prep-HPLC was used to further purify tricin. The purity of tricin reached 99.4%, with a recovery yield of 78.0% thereafter. Tricin exerts an inhibitory effect on the proliferation of various tumor cells, including gastric cancer SGC-7901 cells. It significantly suppresses cell colony formation while also altering cell cycle progression metabolism by decreasing the proportion of cells in the G_0_/G_1_ phase and increasing the proportion in the S and G_2_/M phases. Additionally, tricin affects the efficiency of SGC-7901 cell lactate production, ATP content, and glucose uptake. These findings suggest that tricin may impede tumor cell proliferation through its impact on cell cycle progression and energy metabolism.

## 1. Introduction

Carex Meyeriana Kunth (CMK), a perennial herb belonging to the Cyperaceae family, is most abundant in the Changbai Mountains of Northeast China [1]. Previous studies have found that cellulose is abundant in CMK [2] and is used as a raw material for the production of cellulose nanocrystals. CMK volatile oil showed good antibacterial activity [3]. Studies on CMK polysaccharides have shown that they possess in vitro antioxidant capacity [4] and immunomodulatory activity [5]. Although some studies have been conducted on CMK, the research content is limited, and further exploration of the medicinal value of CMK is necessary. Specifically, there is a need to improve the efficiency of extraction, separation, purification, and analysis of the effective components. Macroporous adsorption resin (MAR) has been widely used in the separation and purification of the active components of different herbal extracts in traditional Chinese medicine, such as flavonoids [6,7], phenolic compounds [8,9], glycosides [10], lipopeptides [11], etc. We used MAR to extract and separate the active components of CMK; tricin (TRI) was also found in CMK.

TRI (5, 7, 4-trihydroxy-3, 5-dimethoxyflavone) is a bioactive flavonoid compound with free and conjugated forms, which is easily soluble in polar solvents, such as methanol, ethanol, and ethyl acetate. Methanol and ethanol are often used as solvents for TRI extraction due to their relatively low toxicity and low environmental pollution [12,13,14]. TRI has significant biological activities against human cytomegalovirus [15] and xanthine oxidase inhibitor [16] and has antioxidant [17] and anti-inflammatory activities [18]. In addition, TRI has shown some unique biological activities, such as anti-leishmanial biological activity [19], anti-obesity effects [20], promoting glucose uptake in C2C12 myotubes [21] and anti-influenza virus activity [22]. With the extensive study of signaling pathways, researchers have found that TRI can effectively improve non-alcoholic fatty liver disease through the AMPK pathway [23]. It promotes autophagy degradation and dopamine release and improves cognitive and motor deficits, as shown in [24] in relation to Parkinson’s disease. TRI can also reduce the development of severe pneumonia induced by lps in bronchial epithelial cells through AKT and MAPK signaling pathways [25] and reduce the continuous development of cerebral ischemia–reperfusion injury through the Sestrin2/Nrf2 signaling pathway [26]. The anti-inflammatory effect of TRI is mainly exerted through the PI3K/Akt and NF-κB signaling pathways [27,28].

Tumors have always had significant impacts on human health and brought great pain to patients. TRI also has a significant inhibitory effect on tumor activity, as found in cases where TRI blocked tumor cell-induced angiogenesis [29] and inhibited the proliferation and invasion of C6 glioma cells by upregulating microRNA-7-targeting focal adhesion kinase [30]. Further, our previous research work found that, in addition to the above studies, TRI has a variety of tumor cytotoxicity. In particular, it has the ability to inhibit the proliferation of gastric cancer cells, which is prominent and has positive significance for the discovery and research of new functions of TRI.

Based on the above context, a method combining MARs and preparation–high-performance liquid chromatography (pre-hplc) was developed to extract and purify TRI from CMK in this study. The adsorption and desorption properties of TRI on six different resins were investigated. The adsorption kinetics and isotherms were also studied, and the adsorption process was fitted to evaluate the effect of the different models. To further investigate the mechanism of the inhibitory effect of TRI on tumor cell proliferation, we carried out in vitro tumor cell proliferation inhibition experiments. This can improve the wider application of CMK and TRI and accelerate the development of new products.

## 2. Results and Discussion

### 2.1. Screening of Adsorption Resins

**Figure 1:**
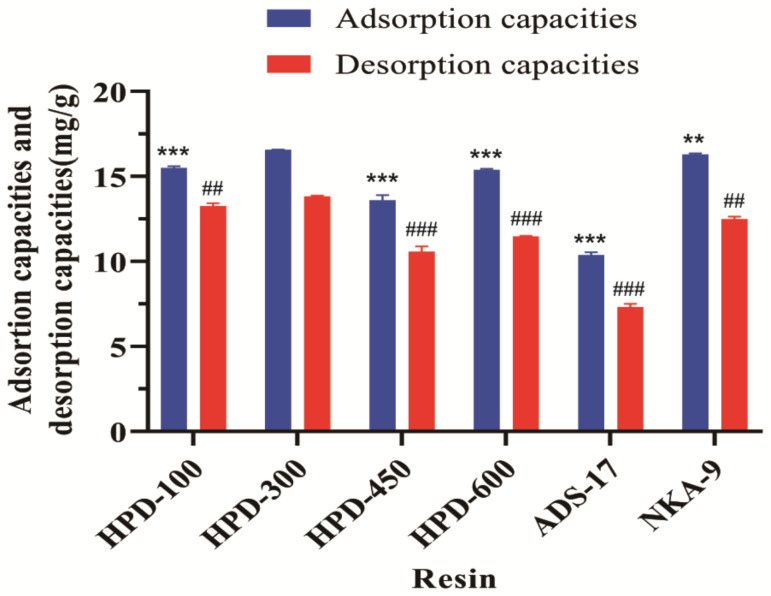
Adsorption/desorption capacities and desorption ratios of TRI on different resins. Data were derived from three independent experiments and are expressed as the mean ± SD. ** *p* < 0.01, *** *p* < 0.001 vs. adsorption capacities of HPD-300; ^##^ *p* < 0.01, ^###^ *p* < 0.001 vs. desorption capacities of HPD-300.

The selection of the most suitable resin for the extraction of flavonoid compounds was a crucial step in our research. We considered various factors, such as adsorption and desorption capacities, to ensure that the chosen resins would effectively capture the target compounds [31]. It was essential to use resins with different polarities due to the complex nature of TRI’s composition. We decided to test six different resins with varying polarities to determine their effectiveness in capturing TRI. Figure 1 depicts the results of our tests, showcasing the adsorption and desorption capacities of each resin for TRI. These data provided valuable insights into which resin would be most suitable for extracting flavonoid compounds from our samples. The adsorption and desorption capacities of moderately polar resins (HPD-450 and ADS-17) were significantly lower than that of other resins, which may be directly related to the weak binding ability of both polar and non-polar groups of the target material and the limited ability to generate hydrogen bonding and van der Waals forces.

The adsorption capacity of polar resin (HPD-600, NKA-9) is also higher; the poly-hydric structure of TRI strengthens the binding between TRI and resin and shows better adsorption capacity; however, its desorption capacity is weaker, which may be because the elution ability of the eluent is be limited by the stronger binding force between the target substance and the resins.

The adsorption/desorption capacities of HPD-100 and HPD-300 resins were better than those of other resins, and no significant differences were found between them in that regard. The comparison of the specific surface areas of the resins showed that of HPD-300 to be greater than that of HPD-100; the average pore diameter was larger in the HPD-100 resin, and the adsorption capacity of HPD-300 was higher due to its higher specific surface area, which provides more adsorption sites; therefore, HPD-300 resin was selected for the subsequent experiments.

### 2.2. Results of Adsorption Kinetics

**Figure 2:**
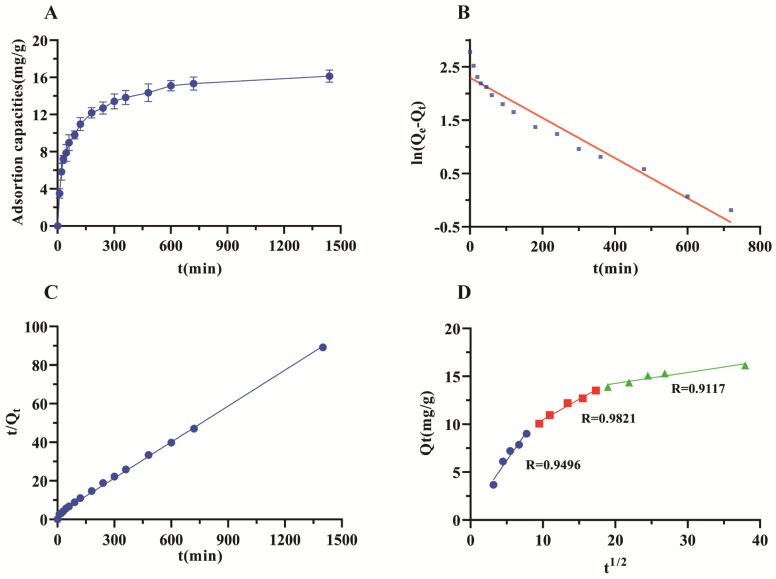
Adsorption kinetic curve and kinetic models for TRI on HPD-300 resin at 25 °C. (**A**): adsorption kinetic curve. (**B**): pseudo-first-order model. (**C**): pseudo-second-order model. (**D**): intra-particle diffusion kinetic model. Q_e_: The equilibrium adsorption capacity based on the dry resin. Q_t_: The adsorption capacity at time t based on the dry resin.

The adsorption kinetics of TRI on the HPD-300 resin are shown in Figure 2A. Generally, the adsorption capacity of adsorption on the adsorbent increased with adsorption time before reaching adsorption equilibrium: the adsorption capacity of TRI increases linearly in the first 120 min, and adsorption capacity shows a greater linear increase, which may be due to the complex composition of the CMK extract and other components competing to occupy the active site of the resin, reducing the rate of adsorption of TRI. The TRI adsorption capacity increased slowly after 120 min and reached equilibrium at 600 min.

To better understand the adsorption mechanism, pseudo-first-order (4), pseudo-second-order (5), and intra-particle diffusion kinetic (6) models were used to reveal the adsorption process. Figure 2B–D show the experimental data with the fitting of the three kinetics models at 25 °C, respectively. Table 1 lists the parameters of the corresponding models. The correlation coefficients of the pseudo-first-order and second-order dynamics models are 0.9468 and 0.9975, respectively. The kinetic parameters obtained from the pseudo-second-order model for TRI (Q_e_ = 21.4592 mg/g) were also close to the experimental data (Q_e_ = 16.1420 mg/g). The results indicate that the pseudo-second-order kinetic model can be used to describe the adsorption kinetics of TRI on HPD-300 resin. The fitting curve of the intra-particle diffusion kinetic model is shown in Figure 2D. The adsorption process is in line with that reported for similar resins and presents several linear processes [32,33]. The initial stage entailed boundary diffusion, the second stage was one of gradual adsorption, and the last stage saw the specimens reach equilibrium. The plot did not pass through the origin, and this indicated that the adsorption process of TRI was affected by both boundary layer diffusion and intra-particle diffusion.

### 2.3. Adsorption Thermodynamics

**Figure 3:**
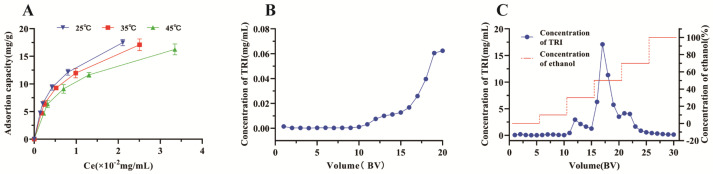
Adsorption thermodynamic and parameters of dynamic for TRI on HPD-300 resin. (**A**): adsorption isotherms at 25, 35, and 45 °C. (**B**): dynamic leakage curve. (**C**): dynamic desorption curve. C_e_: the equilibrium concentration of TRI in the solution.

The adsorption isotherms provide a fundamental understanding of the interaction between an adsorbent and an adsorbate at equilibrium. They illustrate how the adsorption capacity of the adsorbent changes with varying concentrations of the adsorbate in the liquid phase while keeping the temperature constant. This relationship is crucial for designing and optimizing processes for separation techniques. By studying these isotherms, we can determine the most efficient conditions for achieving the maximum adsorption capacity. In this study, 50 mL solutions of TRI (0.049–0.196 mg/mL) were used for the adsorption thermodynamics experiment. The adsorption resin was HPD-300, and the adsorption temperatures were 25 °C, 35 °C, and 45 °C. The adsorption thermodynamic curve is shown in Figure 3A. The adsorption capacity of TRI on HPD-300 resin decreased with rising temperature, indicating that the adsorption of TRI is an exothermic process, which was identical to most of the adsorption processes in the resins [11,32]. The fitting equation and correlation coefficient are listed in Table 2. From the regression analysis, the 1/n values for TRI using the Freundlich equation were between 0.1 and 0.5, indicating the effective adsorption of TRI on HPD-300 [34]. However, the correlation coefficient R^2^ of the Langmuir equation (0.9908–0.9941) was found to be higher than that of the Freundlich equation (0.9776–0.9823), suggesting a monolayer-adsorption process.

### 2.4. Dynamic Breakthrough Curves

The breakthrough curve of TRI on HPD-300 resin was investigated using a dynamic test to determine the optimal loading of CMK extract. According to the literature [11], the leakage point was considered when the initial concentration of 10% was detected in the eluent during the adsorption process. As shown in Figure 3B, the leakage point began to appear when the eluent reached 16 BV; then, the leakage rate increased linearly thereafter. Therefore, 16 BV was selected as the appropriate loading volume.

### 2.5. Dynamic Desorption Curve

Adsorption and desorption is a dynamic process in which the dissolution solution and the resin compete to adsorb the target substance. The optimization of the process parameters has a significant role in the whole experiment. A series of experiments were conducted to obtain dynamic desorption curves to illustrate the relationship between the volume of desorbed liquid and the concentration of TRI in the desorbed liquid. Firstly, the resin was saturated with TRI adsorption using a CMK extraction saturated chromatography column. Unadsorbed impurities and weakly adsorbed materials were removed from the resin surface with 5 BV of deionized water. This cleaning step is essential to prepare the resin for the subsequent desorption process. The desorption process was then solved using different concentrations of aqueous ethanol solutions, with each solvent volume equal to 5 BV, following the elution process shown in Figure 3C. The desorption solutions collected at each ethanol concentration were analyzed to determine the concentration of TRI. These data were used to construct dynamic desorption curves. As shown in Figure 3C, most of the TRI in the resin can be desorbed by 30%, 50%, and 70% ethanol in water (*v*/*v*). Therefore, the appropriate desorption process was run as follows: firstly, 5 BV of deionized water and 5 BV of 10% ethanol aqueous solution (*v*/*v*) were used to remove impurities; TRI was then eluted with 5 BV of 30%, 5 BV of 50%, and 5 BV of 70% ethanol in water (*v*/*v*), and the eluent was collected.

**Figure 4:**
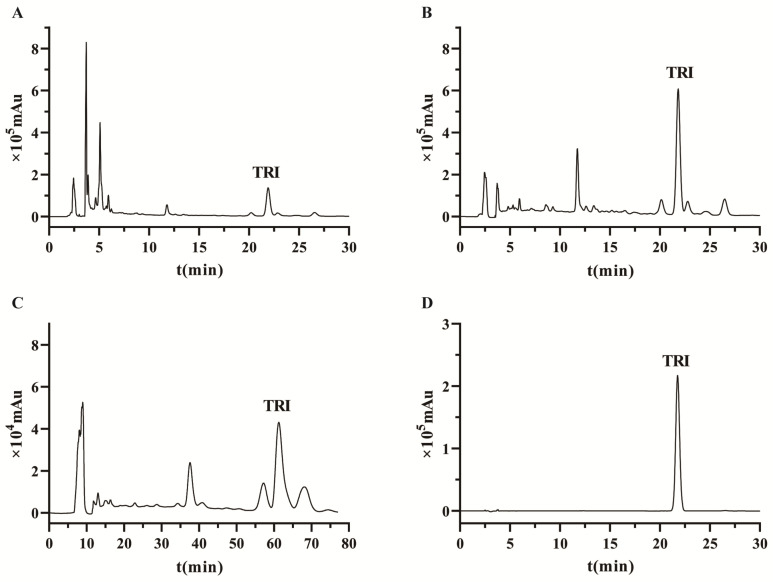
HPLC chromatogram of CMK extracts purification using HPD-300 resin. (**A**): the chromatography before purification using MAR. (**B**): the chromatography after purification using MAR. (**C**): Prep-HPLC chromatogram of TRI. (**D**): the chromatogram of TRI in the final product.

The eluents (30%, 50%, and 70%) were sufficiently dried, and the content of TRI was determined using HPLC. The HPLC chromatographs (before and after purification) are displayed in Figure 4A,B. The relative content of TRI was significantly increased, and the content of TRI was increased from 2.6 mg/g to 45.1 mg/g with a recovery yield of 76.4%.

### 2.6. Prep-HPLC Separation of TRI

The further extraction of TRI was performed using Prep-HPLC. Firstly, the CMK extract (1.0 g) was purified with HPD-300 resin to obtain the preliminary product. Then, the preliminary product was further purified using prep-HPLC according to the peak information (as labeled in Figure 4C). The product was recrystallized in methanol as the solvent. Finally, 380 mg of TRI was obtained with a recovery of 78.0%. As shown in Figure 4D, the purity of TRI could exceed 98% in the final product. The structure of TRI was identified from CMK, as illustrated in Figures S1–S3. These data are consistent with the literature [34,35]; therefore, the compound was firmly identified as TRI.

### 2.7. TRI Inhibited Tumor Cell Activity In Vitro

**Figure 5:**
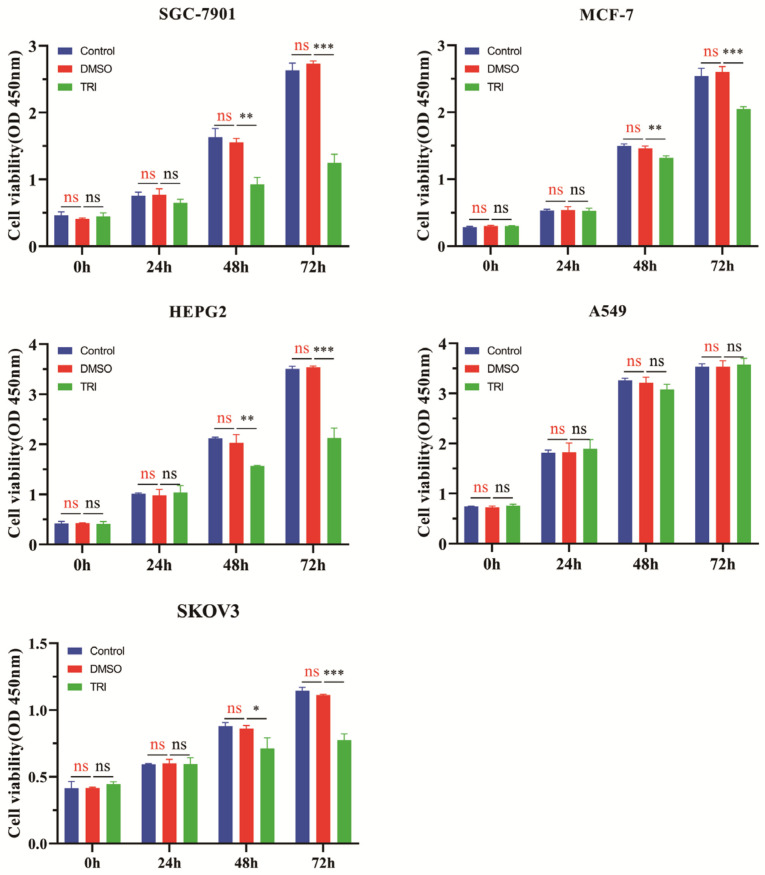
The results of TRI inhibition of 5 tumor cells. Cell viability was evaluated as described in Section 3 and is expressed as OD (450 nm). The experimental process was set up as a control group (DMSO: 0%, TRI: 0 μg/mL), the DMSO group (DMSO: 0.05%, TRI: 0 μg/mL), and a TRI group (DMSO: 0.05%, TRI: 20 μg/mL). Data were derived from three independent experiments and are expressed as the mean ± SD. The “ns” marked in red are DMSO groups vs. control groups. The “ns” marked in black, * *p* < 0.05, ** *p* < 0.01, *** *p* < 0.001, are TRI groups vs. DMSO groups.

The cell viability experiments to assess the effect of TRI on tumor cells were assessed using various human tumor cell lines, including HEPG-2, MCF-7, SGC-7901, and SKOV3, in vitro. CCK8 cell viability assays were conducted to evaluate this effect. The experimental groups were control (DMSO: 0%), DMSO (DMSO: 0.05%), and TRI (DMSO: 0.05%). As shown in Figure 5, there were no significant differences in cell viability between the control group and the DMSO group. Such experimental results suggested that the addition of 0.05% DMSO did not have an obvious effect on cell viability, and the 0.05% DMSO did not show obvious cytotoxicity. In order to ensure the consistency of the system in the subsequent experiments, 0.05% DMSO was added to the control group and TRI group to ensure the uniformity of the system.

**Figure 6:**
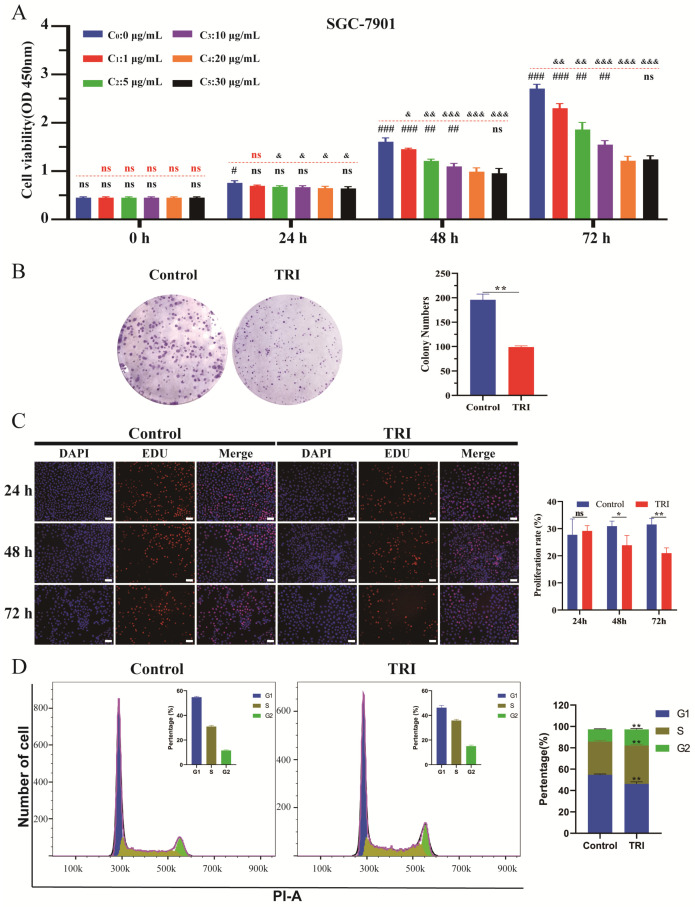
The results related to cell–cell viability. (**A**): TRI inhibited the cell viability of SGC-7901 cells. Cell viability was evaluated as described in Section 3 and is expressed as OD (450 nm). DMSO (0.05%) was added to each experimental group, and TRI concentration was different among the groups (C_0_: 0 μg/mL, C_1_: 1 μg/mL, C_2_: 5 μg/mL, C_3_: 10 μg/mL, C_4_: 20 μg/mL, C_5_: 30 μg/mL). Data were derived from three independent experiments and are expressed as the mean ± SD. The “ns” marked in red, ^&^ *p* < 0.05, ^&&^ *p* < 0.01, ^&&&^ *p* < 0.001 vs. C_0_; The “ns” marked in black, ^#^ *p* < 0.05, ^##^ *p* < 0.01, ^###^ *p* < 0.001 vs. C_4_. (**B**): image of colony formation of SGC-7901 cells. (**C**): figure of the results of the EdU assay to detect the proliferation ability of SGC-7901 cells. The scale: 50 μm. (**D**): glow cytometric analysis of SGC-7901 cell cycle distribution-treated. In Figure (**B**–**D**), the experimental groups are the control group (DMSO: 0.05%, TRI: 0 μg/mL) and the TRI group (DMSO: 0.05%, TRI: 20 μg/mL), * *p* < 0.05, ** *p* < 0.01 vs. control; the data were derived from three independent experiments and are expressed as the mean ± SD.

As shown in Figure 5, TRI showed a significant inhibitory effect on cell viability in a variety of tumor cell lines in a significant time-dependent manner, except for A549 cells, for which the inhibitory effect was insignificant. In addition, Figure 6A shows that the inhibitory effect of TRI on SGC-7901 cell viability was time-dependent, with an IC50 of 53.8 μg/mL and 17.8 μg/mL at 48 h and 72 h, respectively. This indicates that cell viability was significantly inhibited with increasing time. Cell viability was also affected by the concentration of TRI. At a low concentration, the cell viability and concentration were dependent, but no significant differences were observed when the concentration was higher than 20 μg/mL. At 48 h and 72 h, there was no significant difference in cell viability between the concentration of 20 μg/mL and 30 μg/mL.

The SGC-7901 cells were extensively analyzed, and as depicted in Figure 6B, the significant inhibition of the cell clone formation assay results was observed upon treatment with TRI. In comparison to the control group, a conspicuous reduction in the number of colonies formed (** *p* < 0.01) was evident, indicating the effective suppression of SGC-7901 cell proliferation by TRI.

5-Ethynyl-2’-deoxyuridine (EdU), a thymidine analog, can be incorporated into replicating DNA molecules instead of thymidine during cell proliferation. The swift and precise assessment of cellular DNA replication activity relies on the specific reaction of EdU with fluorescent dyes, enabling rapid evaluation of cell proliferation ability. As shown in Figure 6C, there was no significant difference in the positive rate of EdU-labeled cells between the TRI group and the control group after 24 h of drug administration, indicating that TRI did not significantly interfere with the DNA replication process of tumor cells after a short period of drug intervention, while there was a significant difference between the two groups after 48 h and 72 h of drug administration (* *p* < 0.05, ** *p* < 0.01). Moreover, this difference increased with the increase in time. The above results indicate that TRI affects the DNA replication process of tumor cells and can inhibit the proliferation of tumor cells.

Alterations in cell cycle dynamics exert a pivotal influence on the regulation of cellular proliferation. Comparative analysis revealed significant changes in the cell cycle profile relative to the control group: there was a pronounced reduction in the proportion of cells residing in the G0/G1 phase (** *p* < 0.01), coupled with a notable increase in the proportions of cells residing in the S and G2/M phases (** *p* < 0.01) (Figure 6D). In the regulation of the cell cycle, the relationship and regulatory mechanism of the G0/G1, S and G2/M phases are crucial. The promotion of the G0/G1 phase usually indicates that the cell is active during the initial stages of growth and division. The arrest of the S and G2/M phases means that cells are inhibited in the process of DNA replication and cell division. The arrest in the S phase may be related to DNA damage, lack of essential nucleotides, or abnormalities in other intracellular signals. The arrest of the G2/M phase is equally important and is often the last check performed by cells before they are ready for division. If the cell finds DNA damage or other abnormalities in the G2 phase, it prevents entry into the M phase to avoid passing defective genetic information to the daughter cell [36,37]. These observations suggest that TRI may induce cell cycle arrest, thereby impeding the growth of SGC-7901 cells. Changes in each stage of the cell cycle are regulated by different proteins, such as P21, CycD/CDK4, Rb, CycA/CDK2, CycB/CDK1, etc. [38]. These proteins are key proteins in the cell cycle pathway, and all of them are involved in multiple processes of the cell cycle. Changes in the expression of a single protein will cause a chain reaction, and the joint action of multiple proteins will produce the result of promoting or inhibiting the cell cycle. Our results demonstrated that TRI induced cell cycle changes, arrested DNA replication and cell division, and significantly inhibited cell viability in SGC-7901 cells. Next, we will continue to investigate the changes in these key proteins induced by TRI and reveal the molecular biological mechanism.

**Figure 7:**
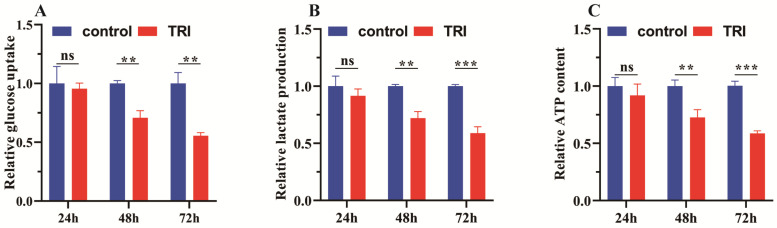
The results of relevant indicators during aerobic glycolysis in SGC-7901 cells treated with TRI. (**A**): relative content of glucose uptake level. (**B**): relative content of lactate. (**C**): relative content of ATP production. The experiment was carried out as described in Section 3. The data were derived from three independent experiments and are expressed as the mean ± SD. The experiment was divided into a control group (DMSO: 0.05%, TRI: 0 μg/mL) and a TRI group (DMSO: 0.05%, TRI: 20 μg/mL), ns indicates no significant difference between the control and TRI groups, ** *p* and *** *p* indicate that the significant difference between the control and TRI groups is significant and statistically significant. ** *p* < 0.01, *** *p* < 0.001 vs. control.

Under the conditions of adequate oxygen availability, tumor cells predominantly reply on aerobic glycolysis to fulfill their energy requirements, setting them apart from normal cells. This phenomenon is commonly known as the Warburg effect. This metabolic adaptation directly impacts the energy metabolism of cancer cells, influencing their proliferation and migration. In this study, we investigated differences in glucose uptake rate, lactate production, and ATP generation during aerobic glycolysis in SGC-7901 cells. Our findings indicate that compared to the control group, there was no significant difference in glucose uptake efficiency, ATP production and lactate production at 24 h (ns) (Figure 7). But as time goes on, the TRI-treated group exhibited a significant decrease in glucose uptake efficiency over prolonged durations (** *p* < 0.01) (Figure 7A), suggesting that TRI may substantially impede glucose uptake in tumor cells. Furthermore, lactate production exhibited significant differences relative to the control group (** *p* < 0.01, *** *p* < 0.001) (Figure 7B), indicating that TRI treatment may attenuate lactate production during aerobic glycolysis. Additionally, our results demonstrated a significant reduction in ATP production (** *p* < 0.01, *** *p* < 0.001) (Figure 7C) in the TRI-treated group compared to the control group, suggesting that TRI may hinder ATP production in tumor cells by modulating aerobic glycolysis. Overall, these findings suggest that TRI treatment disrupts the energy metabolism pathway of tumor cells, potentially inhibiting cell proliferation by suppressing the Warburg effect. These results offer valuable insights into potential therapeutic avenues targeting cancer cell metabolism and underscore the need for further elucidation of the mechanisms underlying TRI’s effects on aerobic glycolysis in tumor cells.

## 3. Materials and Methods

### 3.1. Materials and Reagents

CMK specimens were collected from Panshi, China, air-dried and ground into powder. Ethanol and methanol were of analytical grade. Acetonitrile was of HPLC grade. The water was ultra-pure water. The apoptosis detection kit with annexin V-FITC and the cell cycle analysis kit were purchased from the Beyotime Institute of Biotechnology (Haimen, China). The Cell-Light^TM^ EdU Apollo in vitro Kit was purchased from Guangzhou Ribo of Bio-technology (Guangzhou, China). The ATP test kit, glucose test kit, and lactic acid test kit were purchased from the Nanjing Jiancheng Bioengineering Institute (Nanjing, China).

### 3.2. Adsorbents

These MARs (HPD-100, HPD-300, HPD-450, HPD-600, ADS-17, and NKA-9) were sourced from Cangzhou Bonchem Co., Ltd. (Hebei, China). Detailed information on the physical properties of these resins is shown in Table 3. In preparation for use, the resins were first soaked in ethanol for 24 h. Then, the resin was transferred to a pre-prepared glass column.

The next step is to thoroughly rinse the resin with deionized water to ensure that the ethanol is completely removed. This is essential to eliminate any residual solvent that may interfere with subsequent processes. After the ethanol was completely washed out, the resin was treated with a 5% *w*/*v* sodium hydroxide (NaOH) solution. This alkaline treatment lasted for 4 h. After the NaOH treatment was complete, the resin was washed again with deionized water until the filtrate had a neutral pH, indicating that there was no residual NaOH.

Subsequently, the resin was soaked in 5% *w*/*v* hydrochloric acid (HCl) solution for 4 h for acid treatment. This step is essential to neutralize the residual alkalis and to further prepare the resin. After the acid soak, the resin was rinsed with deionized water until the pH of the filtrate returned to neutral (pH 7). This series of treatments ensures that the resin is thoroughly cleaned and prepared for its intended application.

### 3.3. Preparation of CMK Extracts

The CMK extracts were prepared using the following methods: 250 g of the dried CMK powders was extracted with 5000 mL of 70% *v*/*v* aqueous ethanol for 90 min via reflux, and the extraction process was repeated twice. After extraction, the extraction solutions were combined, and the ethanol and a portion of water were volatilized at 40 °C under reduced pressure to obtain CMK crude extract (TRI: 0.196 mg/mL).

### 3.4. Determination of TRI Content

The experimental procedure was carried out according to the literature, and the mobile phase types and ratios were optimized appropriately. The optimized approach: HPLC analysis was conducted using an LC-20AT system (Shimadzu, Japan) equipped with a Dikma C_18_ column (4.6 × 250 mm, 5 μm). Acetonitrile (A) and 0.1% phosphoric acid (B) were used as the mobile phase (A:B = 37:63) with a flow rate of 1.0 mL/min, and the whole process involved isocratic elution. The detection wavelength was set to 350 nm, the injection volume was 10 μL, and the column temperature was set to 40 °C. The regression line of TRI was A = 89,002C + 190,970 with good linearity (R^2^ = 0.9996), where A represents the peak area and C is the concentration (μg/mL) of TRI.

### 3.5. Screening of Adsorption Resins by Static Adsorption/Desorption Experiments

Each of the pre-treated hydrated resins (the dry mass of the resin was 0.5 g) could be placed into a 100 mL conical flask with a stopper, and then 50 mL of CKM crude extract solution was added. All of the flasks were placed in a constant temperature shaker (120 rpm) and shaken for 12 h at 25 °C. During this process, the chemical constituents in the extracts compete dynamically between the resin and the desorbed solvent. With sufficient time, this equilibrium reaches a steady state where the adsorption sites inside and on the surface of the resin are fully saturated, and the whole adsorption process is complete. The resin and the extract solution were separated, and the resin was washed with a small amount of deionized water; the extract solution and deionized water were collected, and the final volume was adjusted to 50 mL. The TRI was analyzed, and the adsorption capacity of the resin was calculated.

After achieving adsorption equilibrium, the resin was subjected to desorption experiments, which were carried out in a constant temperature shaking shaker (12 h, 25 °C, and 120 rpm), and the desorption solvent was 50 mL of 85% *v*/*v* ethanol solution. After desorption, the resolving solution was separated, and the volume of 85% *v*/*v* ethanol solution was fixed to 50 mL. The content of TRI in the resolving solution was analyzed, and the desorption capacity was calculated.

The adsorption/desorption capacities and desorption ratios of the resins were quantified according to the equations provided.

(1) $$\mathrm{Adsorption}\;\mathrm{capacity}:Q_{e}=\frac{(C_{0}-C_{e})V_{0}}{W}$$

(2) $$\mathrm{Desorption}\;\mathrm{capacity}:\;Q_{d}=\frac{C_{d}V_{d}}{W}$$

(3) $$\mathrm{Desorption}\;\mathrm{ratio}\;(\%):\;D=\frac{C_{d}V_{d}}{\left(C_{0}-C_{e}\right)V_{0}}\times 100\%$$

where Q_e_ is the equilibrium adsorption capacity based on the dry resin (mg/g); C_0_ and C_e_ are the original and equilibrium concentrations of TRI in the solution, respectively (mg/mL); V_0_ is the volume of the initial sample solution (mL); W is the mass of the resin free of moisture (g); Q_d_ is the desorption capacity based on the dry resin(mg/g); Dis the desorption ratio (%); C_d_ is the concentration of TRI in the desorption solution (mg/mL); and V_d_ denotes the volume of the desorption solution (mL).

### 3.6. Adsorption Kinetics

An adsorption kinetic study was undertaken according to the method described elsewhere [32] with slight modifications. Pre-treated resin 0.5 g was added to a 100 mL conical flask, and 50 mL of aqueous crude extract was added and mixed thoroughly, which was carried out in a constant temperature shaking shaker (24 h, 25 °C, and 120 rpm), 0.5 mL of the resolving solution was aspirated at different time points, as shown in Figure 2A, and the content of TRI was determined using HPLC. The mathematical description of the adsorption phenomenon was calculated according to the following model, Equation (9):

(4) $$\mathrm{Pseudo-first-order}\;\mathrm{kinetics}\;\mathrm{model}:\;\ln\left(Q_{e}-Q_{t}\right)=lnQ_{e}-K_{1}t$$

(5) $$\mathrm{Pseudo-second-order}\;\mathrm{kinetics}\;\mathrm{model}:\;\frac{t}{Q_{t}}=\frac{1}{K_{2}Q_{e}^{2}}+\frac{t}{Q_{e}}$$

(6) Particle diffusion kinetics model: Qt=Kit12+C

where Q_e_ and C_e_ are set as specified in Equation (1). Q_t_ denotes the adsorption capacity at time t based on the dry resin (mg/g); K_1_, K_2_, and K_i_ are rate constants for the pseudo-primary, pseudo-secondary and particle diffusion adsorption kinetic models, respectively. C is a constant in the dynamic model of particle diffusion.

### 3.7. Adsorption Isotherms

An adsorption isotherm study was undertaken according to the method described in the literature [32] with slight modifications. The adsorption isotherms of TRI on the selected resin were investigated using 50 mL of CMK crude extracts solution at different concentrations with 0.5 g of the resin in a series of 100 mL conical flasks. The flasks were shaken at different temperatures (25, 35, or 45 °C) for 12 h while being shaken at 120 rpm. The equilibrium concentration of TRI was investigated using HPLC. The adsorption phenomenon was mathematically described using the Langmuir equation model and Freundlich equation model.

(7) $$\mathrm{Langmuir}\;\mathrm{equation}:\;\frac{C_{e}}{Q_{e}}=\frac{C_{e}}{Q_{m}}+\frac{1}{Q_{m}K_{L}}$$

(8) $$\mathrm{Freundlich}\;\mathrm{equation}:\;lnQ_{e}=\frac{1}{n}lnC_{e}+lnK_{F}$$

where Q_e_ and C_e_ are set as specified in Equation (1). Q_m_ is the maximum adsorption capacity (mg/g). K_L_ stands for adsorption constant (mg/mL). K_F_ stands for Freundlich constant. $\frac{1}{n}$ is the adsorption empirical constant.

### 3.8. Dynamic Adsorption and Desorption Tests

Dynamic adsorption and desorption were evaluated using a glass column measuring 12 × 300 mm containing 5.0 g of HPD-300 resin. The specie bed volume (BV) was 25 mL. An aqueous solution containing the crude CMK extract was delivered to the HPD-300 resin column at a rate of 2 BV per hour. Subsequently, the column containing the adsorbent was rinsed with 5 BV of deionized water and then eluted with an aqueous ethanol solution at the same 2 BV/h rate. The TRI content in the effluent and eluent was determined using HPLC analysis. Subsequently, the eluate was concentrated to dryness under vacuum to isolate pure TRI.

### 3.9. Purification of TRI using prep-HPLC

Upon treatment with HPD-300 resin, TRI was further separated using a Shimadzu LC-20AR chromatography system. The initially purified samples were dissolved in 50% methanol solution and injected into a Shim-pack GIST C18 column (20 × 250 mm, 5 μm) after completely dissolving for further separation. The mobile phase of the chromatographic system was acetonitrile (A) and 0.05% trifluoroacetic acid (B) (A:B = 30:70) in an isocratic elution, and the flow rate was set at 7 mL/min. The detection wavelength was 350 nm, and the injection volume was 2.0 mL.

### 3.10. Cell Viability Assay and Cell Proliferation Assay

The cells in the logarithmic growth phase were seeded in a 96-well plate at a density of 6 × 10^3^ cells and incubated at 37 °C with 5% CO_2_ for 12 h. A drug-containing solution was added to each well and incubated continuously for 24, 48, or 72 h.

The cell viability assay was inspected using CCK-8 in accordance with the test kit, and 100 μL of CCK-8 detection solution was added to each well and incubated for 2 h away from light. Finally, the absorbance of each well was read with an enzyme meter (at 450 nm), and the OD value was recorded.

The cell proliferation assay was inspected by using a Cell-Light^TM^ EdU Apollo in vitro kit. The 100 μL EdU solution was added to each well and incubated at 37 °C for 2 h. The solution was discarded, fixed with 4% paraformaldehyde for 30 min, and permeabilized with 0.5% triton for 40 min. Apollo staining solution was added and incubated in the dark for 30 min. DAPI was used to stain the nuclei, and the cells were observed under a microscope, photographed, and counted.

### 3.11. Clone Formation Assay

The cells in the logarithmic growth phase were seeded in 6-well plates at a density of 700 cells per well and cultured for 14 days at 37 °C with 5% CO_2_. After completing the experiment, the cells were fixed with a 4% paraformaldehyde solution for 15 min. The cells were incubated with 1% crystal violet staining solution for 10–30 min at room temperature. After incubation, the well plates were photographed to calculate the clone formation rate.

### 3.12. Cell Cycle Assay

Cells in the logarithmic growth phase were seeded in 6-well plates at a density of 4 × 10^5^ cells per well and cultured for 12 h at 37 °C with 5% CO_2_. The drug was added to each well and incubated for 48 h. The experiment was performed according to the kit instructions, and detection was conducted using a flow cytometer.

### 3.13. Cell Culture Protocol for Determination of Glucose, Lactate, and ATP Content

Logarithmic growth phase cell suspensions were seeded in 6-well plates at a standard of 4 × 10^5^ cells per well. Two groups were established: a control group and a drug administration group. The cells were cultured for 24, 48, and 72 h. After culturing, the supernatant was collected in 2 mL volumetric flasks to determine the glucose and lactic acid content. The cells were washed twice with PBS and suspended in 1 mL of PBS. The suspension was extracted using a cell ultrasonic pulverizer to determine the ATP and protein content in the cells.

The glucose assay, lactic acid production, and ATP content were determined using methods that are already reported in the literature [39,40]. The experimental steps were carried out according to the instructions provided in the kit. The results were based on the protein concentration.

## 4. Conclusions

A novel and effective method for the preparation and separation of TRI from CMK was proposed. It used macroporous resin (HPD-300) and prep-HPLC. The kinetic data pertaining to TRI adsorption on HPD-300 resin conformed to the quasi-second order equation. The Langmuir equation was used to describe the adsorption process of TRI on HPD-300 resin. The purity of TRI increased from 2.6 mg/g to 45.1 mg/g, with a recovery yield of 76.4% after purification using HPD-300 resin. The product arising from the HPD-300 resin treatment was separated using high prep-HPLC. The purity of TRI was 99.4%, and the recovery reached 78.0%. This method was deemed suitable for the large-scale purification of TRI from CMK. Studies on the inhibitory activity of TRI in vitro showed that TRI could inhibit the proliferation of tumor cells by affecting the processes of cell cycle progression and energy metabolism. These studies can provide reliable data for the in-depth development of TRI and facilitate the rationalized application of CMK and TRI.

## Figures and Tables

**Table 1:** Parameters of the kinetic models.

Temperature (K)	Langmuir Equation			Freundlich Equation		
	Q_max_	K_L_	R^2^	K_F_	1/n	R^2^
298	19.6078	255.0000	0.9941	111.8770	0.4668	0.9815
308	19.0114	175.3333	0.9928	98.6620	0.4622	0.9823
318	18.1488	137.7500	0.9908	78.4923	0.4474	0.9776

Q_max_: the maximum adsorption capacity. K_L_: the adsorption constant. K_F_: the Freundlich constant. 1/n: the adsorption empirical constant.

**Table 2:** Langmuir and Freundlich adsorption parameters of TRI on the HPD-300 resin at different temperatures.

Model	Equation	R^2^	K_1_	K_2_	Q_e_ (mg/g)
The pseudo-first-order	ln (Q_e_ − Q_t_) = 2.2956 − 0.0038t	0.9468	0.0038	\	9.9304
The pseudo-second-order	t/Q_t_ = 0.0609 + 0.0466t	0.9975	\	0.0356	21.4592

Q_e_: the equilibrium adsorption capacity based on the dry resin. Q_t_: the adsorption capacity at time t based on the dry resin. K_1_: the rate constant of the pseudo-first-order. K_2_: the rate constant of the pseudo-second-order.

**Table 3:** Specifications of the six macroporous resins.

Resin	Polarity	Particle Size (mm)	Surface Area (m^2^/g)	Average Pore Diameter (Å)
HPD-100	Non-polar	0.30–1.20	650–700	85–90
HPD-300	Non-polar	0.30–1.20	800–870	50–55
HPD-450	Moderately polar	0.30–1.20	500–550	90–110
HPD-600	Polar	0.30–1.20	550–600	80
ADS-17	Moderately polar	0.30–1.25	90–150	250–300
NKA-9	Polar	0.30–1.25	500–550	100–120

## Data Availability

The data presented in this study are available upon request from the corresponding author.

## Supplementary Materials

The following supporting information can be downloaded at https://www.mdpi.com/article/10.3390/molecules29194530/s1, S1: Identification of TRI; Figure S1: 13C-NMR characteristic spectra of TRI; Figure S2: H-NMR characteristic spectra of TRI; Figure S3: Mass spectrogram of TRI.
