# Supplementary material for: Preparation and Tumor Inhibitory Activity of Tricin from Carex Meyeriana Kunth
Source: Molecules. 2024 Sep 24;29(19):4530. doi: 10.3390/molecules29194530 (PMC11478251; doi:10.3390/molecules29194530)
Supplement: Supplementary file 1 [file molecules-29-04530-s001.zip › Supplementary Materials.docx]

Supplementary Materials

Preparation and tumor inhibitory activity of tricin from Carex Meyeriana Kunth

Baiji Cui^1,2,†^, Jie Sun^3^, Sheng Chang^2^, Hongmei Zhang^2^, Yawei Li^2^, Xianmin Feng^3,^*, and Zengjun Guo^1,^*

1 School of Pharmacy, Health Science Center, Xi'an Jiaotong University, Xi'an, 710061, China;

2 School of Pharmacy, Jilin Medical University, Jilin, 132013, China;

3 Academy of Basic Medicine, Jilin Medical University, Jilin, 132013, China

* Corresponding author: Zengjun Guo. E-mail address: guozj@mail.xjtu.edu.cn; Xianmin Feng. E-mail address: fengxianmin28@163.com.

Number of pages: 6

Number of figures: 3

List

**S1**. Identification of TRI

**Figure S1.**13C-NMR characteristic spectra of TRI.

**Figure S2.**H-NMR characteristic spectra of TRI.

**Figure S3.**Mass spectrogram of TRI.

**S1.** Identification and Structure of TRI

Nuclear magnetic resonance (NMR) spectra of TRI were recorded on a Bruker AVANCE Ⅲ 600 MHz instrument in methanol-D_4_ (CD_3_OD) using tetramethylsilane (TMS) as the internal standard. TRI was identified by using an Agilent 1260-6120 liquid chromatograph-mass spectrometry system with an optical multichannel diode array detector and a single quadrupole mass spectrometer detector. Electrospray ionization (ESI) was applied in positive ion mode (PI) for the MS analysis. The operating conditions for mass analysis were set as follows: the capillary voltage was 3.0 kV; the drying gas flow was 10.0 L/min; the nebulizer pressure was 30 psi (g); the drying gas temperature was 350 °C. The mass spectra were recorded in the mass range from 100 ≤ m/z ≤ 1000.

TRI was obtained as a pale yellow powder with molecular formula C_7_H_14_O_7_, which was deduced from the ESI-MS m/z: 331.1 [M+H]+. The 1H and 13C-NMR spectroscopic data are as follows: 1H-NMR (600 MHz, CD_3_OD) δ: 6.23 (1H, d, J = 1.2Hz, H-6), 6.49 (1H, d, J = 1.2 Hz, H-8), 6.67 (1H, s,H-3), 7.26 (2H, s, H-2′, 6′), 3.96 (6H, s, 3′, 5′-OCH_3_); 13C-NMR (150 MHz, CD_3_OD) δ: 182.4 (C-4), 164.7 (C-2, C-7), 161.8 (C-5), 158.0 (C-9), 148.3 (C-3′, 5′), 139.8 (C-4′), 121.3 (C-1′), 104.0 (C-2′, 6′), 103.2 (C-3, C-10), 98.8 (C-6), 93.7 (C-8), and 55.7 (2 × OCH_3_).


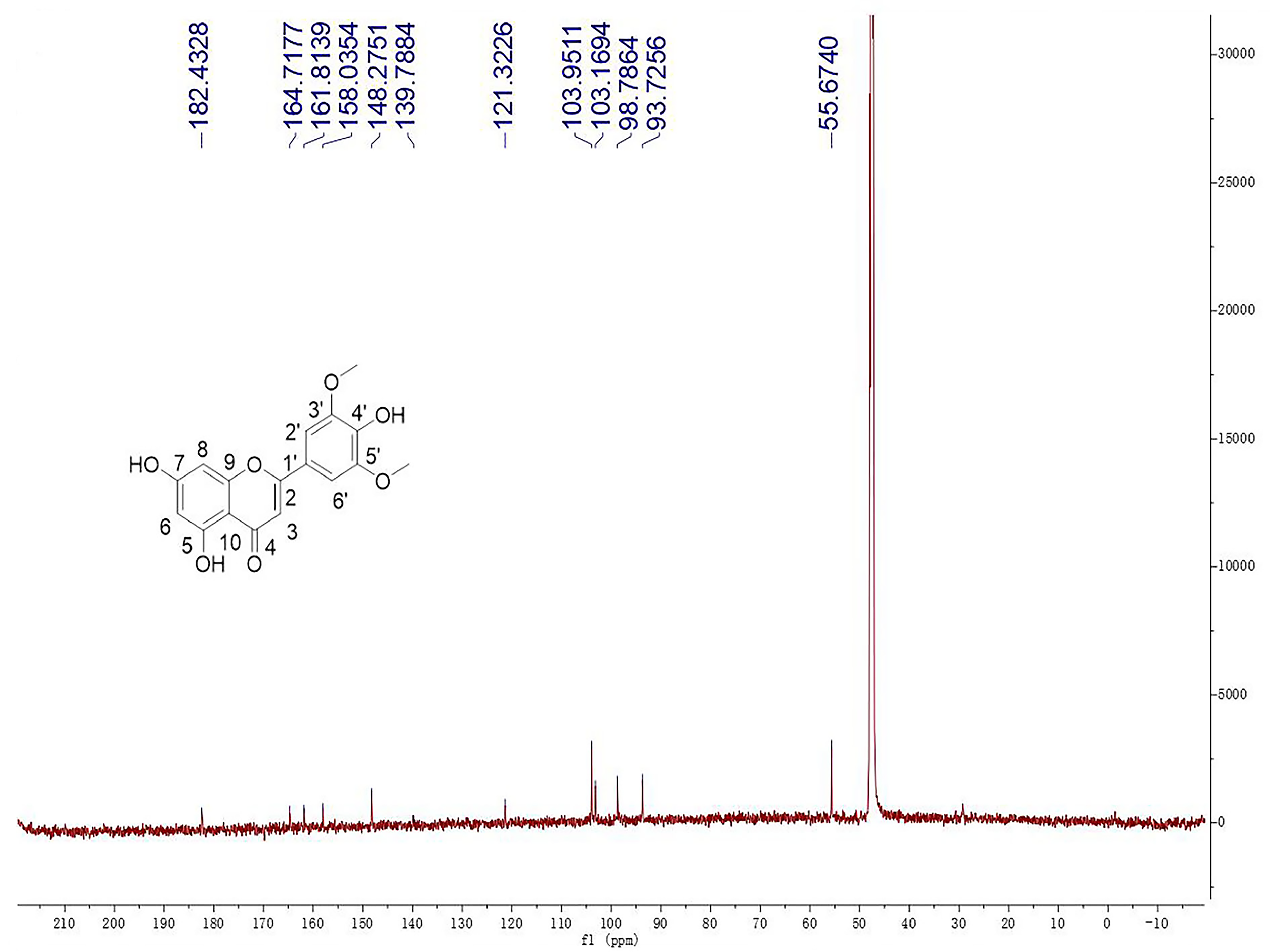


**Figure S1.**^13^C-NMR characteristic spectra of TRI.


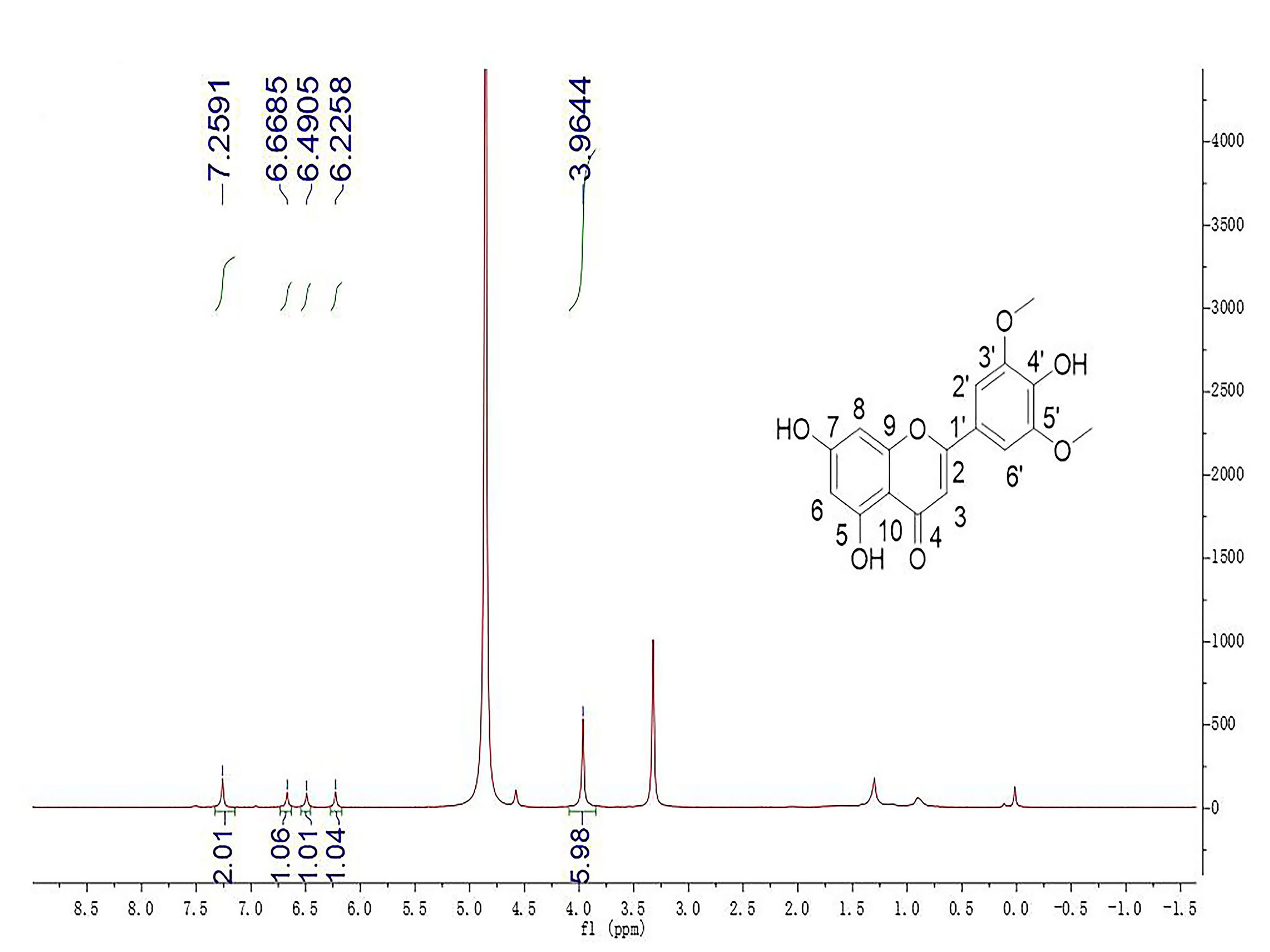


**Figure S2.**H-NMR characteristic spectra of TRI.


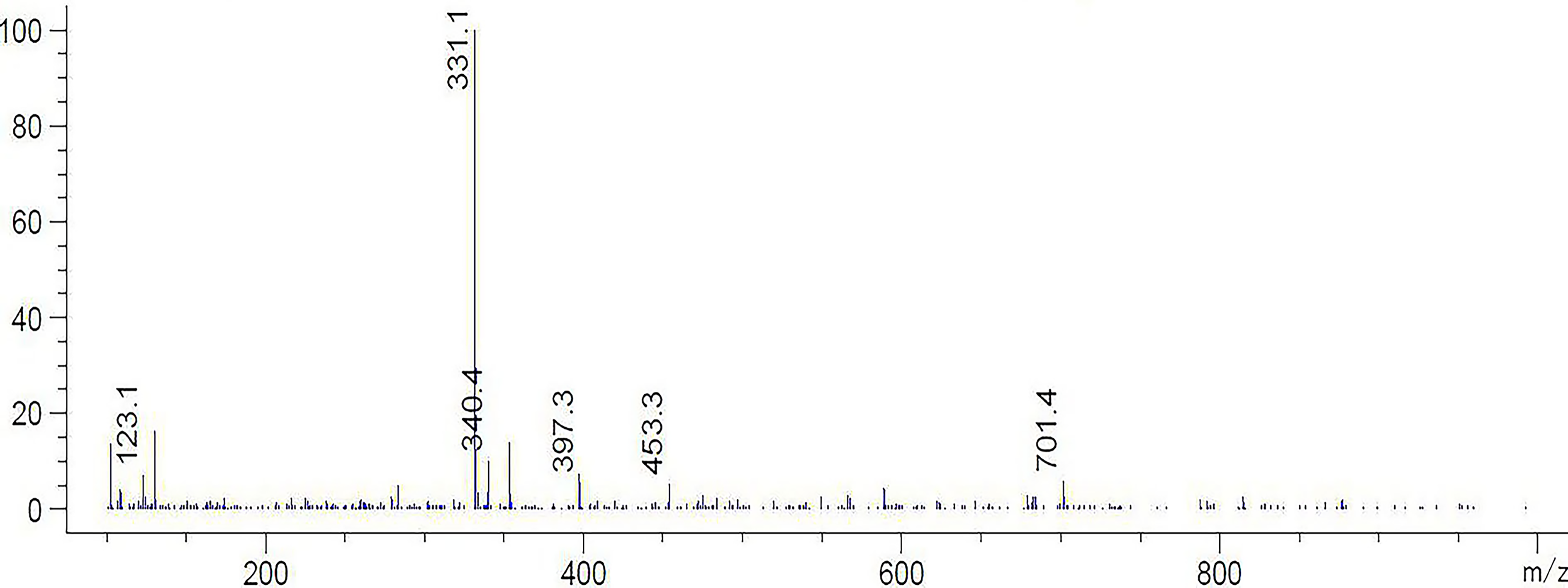


**Figure S3.**Mass spectrogram of TRI.
